# Supplementary material for: Composite fermion liquid to Wigner solid transition in the lowest Landau level of zinc oxide
Source: Nat Commun. 2018 Oct 19;9:4356. doi: 10.1038/s41467-018-06834-6 (PMC6195604; doi:10.1038/s41467-018-06834-6)
Supplement: Supplementary file 1 — Supplementary Information [file 41467_2018_6834_MOESM1_ESM.pdf]

Supplementary Information: Composite fermion liquid to Wigner solid transition in the lowest Landau level of zinc oxide

Maryenko et al.

Supplementary Note 1: $I - V$ characteristics and temperature dependence of insulating phases

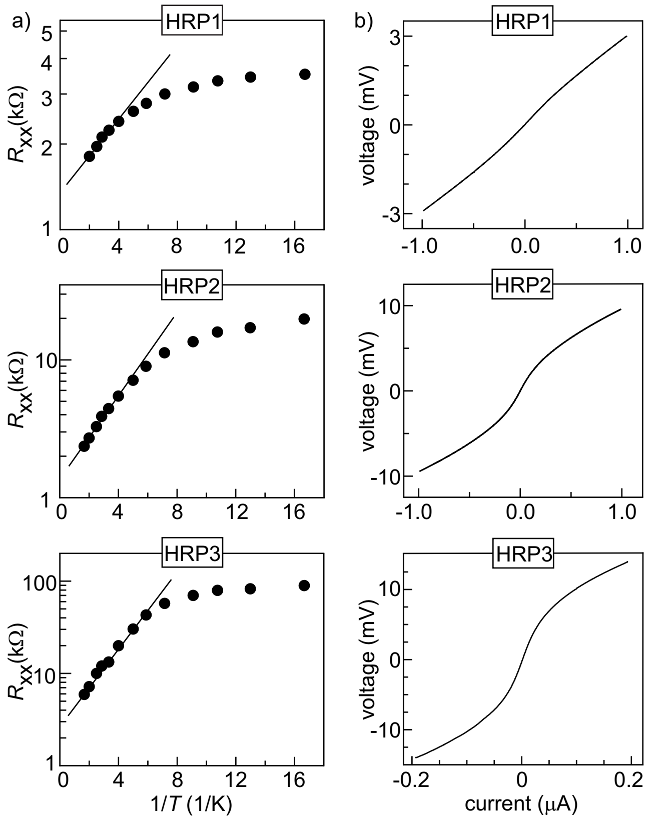

Supplementary Figure 1. **Characteristics of high resistance phases.** (a) Temperature dependence resembles melting behavior of Wigner solid. R_{xx} is taken at the maximum of each high resistance phase indicated as HRP1, HRP2 and HRP3.(b) $I - V$ characteristics acquired at the magnetic field, at which HRP1, HRP2 and HRP3 have the largest R_{xx}.

Here we present some additional data for the $I - V$ characteristics, when the electron system enters high resistance phases, which might be attributed to Wigner solid. Supplementary Fig. 2a shows R_{xx} at several temperatures in the region of formation of high resistance phases HRP1, HRP2 and HRP3. We focus on the transport around filling factor ν=1/3. On both sides of $\nu = 1/3$ one finds a critical field separating metallic region ($\partial R_{xx}/\partial T > 0$) from the insulating region ($\partial R_{xx}/\partial T < 0$). Panel b) below depict $I - V$ characteristics in both metallic and insulating regions, not far from the critical field, acquired at base temperature of the experiment. The character of $I - V$ dependence changes clearly, thus supporting the formation of Wigner solid phase.

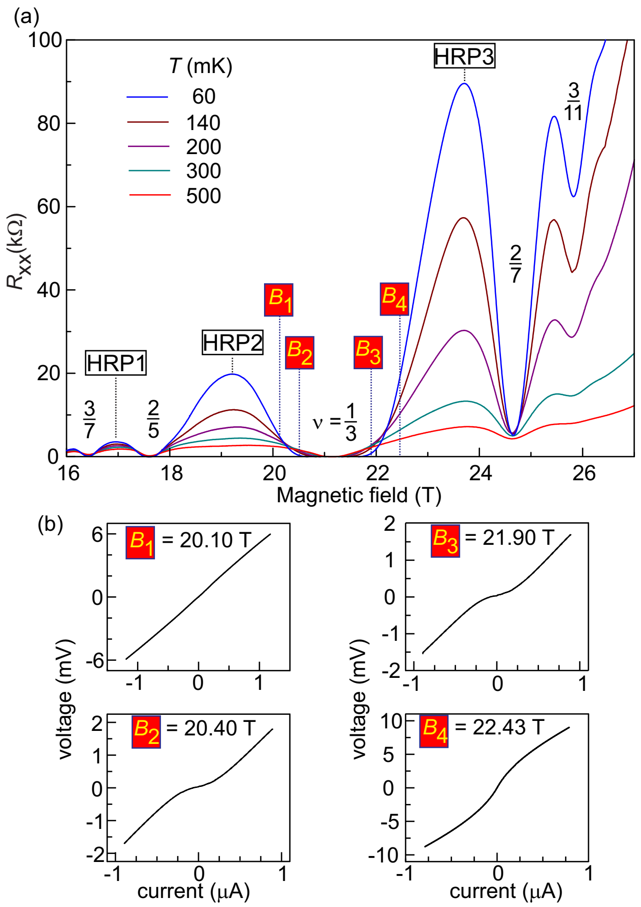

Supplementary Figure 2. **Metal-Insulator Transition around** $\nu = 1/3$**.** (a) R_{xx} versus B near $\nu = 1/3$. Metal-insulator transition occurs between B_1 and B_2 as well as between B_3 and B_4. Metallic region is characterized by $\partial R_{xx}/\partial T > 0$, while the insulator region is characterized by $\partial R_{xx}/\partial T < 0$. (b) $I - V$ characteristics at various magnetic fields indicated in (a). The change of $I - V$ behavior at fields B_1-B_2 and B_3-B_4 correlates with the metal-insulator transition.

Supplementary Note 2: Composite fermion mass

Supplementary Fig. 3 compares the mass of composite fermions extracted for the sample under study with the results of mass analysis in previous studies. A linear dependence of composite fermions mass $m_{\rm CF}$ on the magnetic field B is well observed.

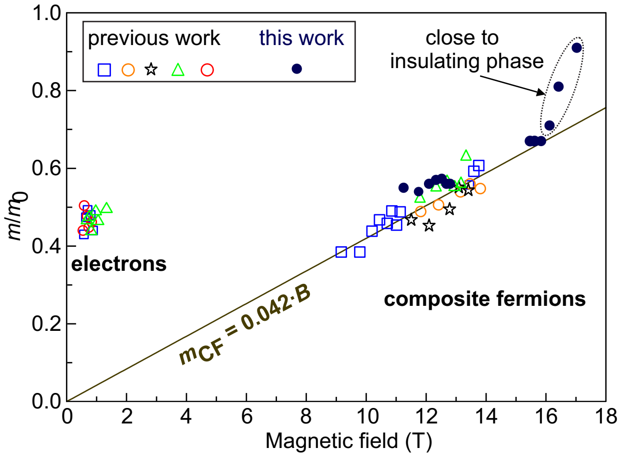

Supplementary Figure 3. **Composite fermions mass.** Mass of composite fermions as a function of magnetic field. Summary of previous samples and the one used in this work. m_0 is the electron free mass.

To estimate the mass of composite fermions we consider that the magnetoresistance oscillations can be described with Lifshitz-Kosevitch approximation and consider here only the leading component of the Taylor expansion. The composite fermions with mass $m_{\rm CF}$ move in an effective magnetic field $B_{\rm eff} = B - B_{\nu=1/2}$. Thus, the oscillatory part of R_{xx} at temperature T is described as:

$$\Delta R_{xx} = R_0 \frac{XT}{\sinh(XT)} \exp\left(-\frac{\pi}{\omega_{\rm CF}\tau_{\rm q}}\right) \tag{1}$$

where $X = \frac{2\pi^2 k_{\rm B}}{\hbar} \frac{m_{\rm CF}}{eB_{\rm eff}}$.

To analyze the mass and the scattering time from the temperature dependence of R_{xx} oscillations amplitude, we take the logarithm of Supplementary Eq.1 and obtain the following equation:

$$\ln(\Delta R_{xx}/T) = \ln(R_0) - \frac{m_{\rm CF}}{eB_{\rm eff}}\frac{\pi}{\tau_{\rm q}} + \ln\left(\frac{2\pi^2 k_{\rm B}}{\hbar}\frac{m_{\rm CF}}{eB_{\rm eff}}\right) - \ln\left(\sinh\left(\frac{2\pi^2 k_{\rm B}}{\hbar}\frac{m_{\rm CF}}{eB_{\rm eff}}T\right)\right) \tag{2}$$

Supplementary Fig. 4 exemplifies the analysis of composite fermion mass at two representative magnetic field values and for four tilt angles θ. It plots $\ln(\Delta R_{xx}/T)$ vs. T (open circles) and the best fit (solid line) obtained using Supplementary Eq. 1. Supplementary Fig. 4a depicts the region $\nu > 1/2$ and demonstrates that the composite fermion mass remains largely unaffected by the application of an in-plane field. Supplementary Fig. 4b depicts $\nu < 1/2$ and demonstrates that the composite fermion mass shows the dependence on the tilt angle. Supplementary Fig. 4c shows that the temperature dependence of R_{xx} oscillation amplitude can also be fitted (solid line) with the Lifshitz-Kosevitch approach. Therefore the composite mass enhancement as discussed in the main text may serves as an indicator for composite fermion localization.

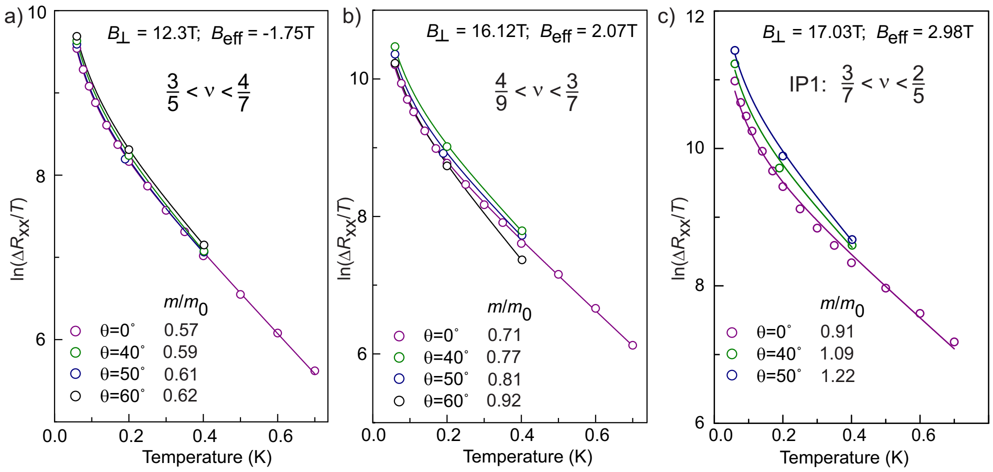

Supplementary Figure 4. **Analysis of composite fermion mass at different tile angles.** a) for ν >1/2. b) $\nu < 1/2$. c) Evaluation of composite fermion effective mass for the phase HRP1.

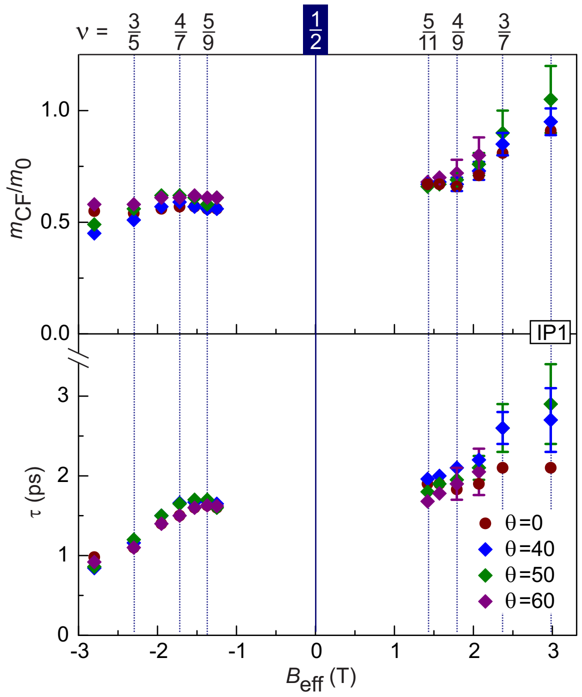

Supplementary Figure 5. **Mass and scattering time of composite fermions.** Composite fermion mass and scattering time of composite fermions at different tilt angles. Error bars represent the standard deviation of the fit parameter using Supplementary Eq. 2.

Supplementary Note 3: Effect of in-plane field on the wavefunction width

The application of the in-plane field squeezes the wavefunction and shifts it towards the interface. The change of the wavefunction width can be estimated by solving the following equations [1, 2]:

$$\frac{b^4}{b_0^4} - \frac{b}{b_0} = \frac{48\tan^2\theta}{(b_0 l_{\rm B})^4} \tag{3}$$

Here, b_0^{-1} is the wavefunction width at zero tilt angle,$l_{\rm B}$ is the magnetic length corresponding to the perpendicular magnetic field component $B_\perp$ and θ is the tilt angle. We solve this equation graphically as shown in Supplementary Fig. 6. The horizontal lines represent the values obtained by calculating the right hand side of the Supplementary Eq. 2 using the parameters shown in the inset of Supplementary Fig. 6 .For the heterostructure studied in this work the wavefunction width is on the order of b_0^{-1}=10nm.

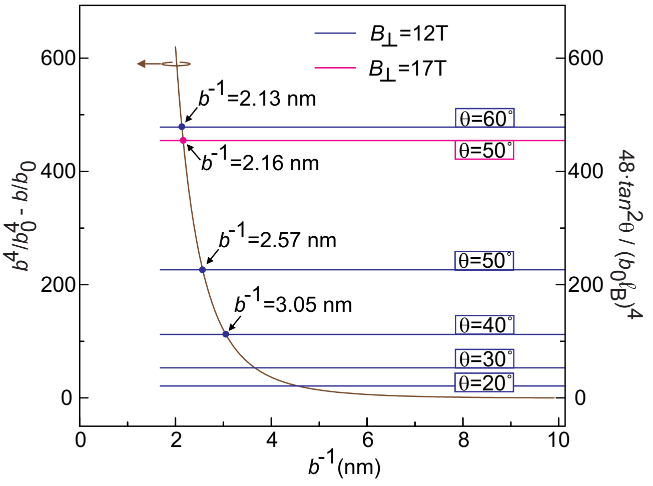

Supplementary Figure 6. **Estimate of wavefunction width.** Calculation of the wave function width due to the application of in-plane magnetic field.

SUPPLEMENTARY REFERENCES

[1] Gee, P. J. *et al.* Composite fermions in tilted magnetic fields and the effect of the confining potential width on the composite-fermion effective mass. Phys. Rev. B **54**, R14313–R14316 (1996).

[2] Chakraborty, T. & Pietiläinen, P Fractional quantum hall effect in tilted magnetic fields. Phys. Rev. B **39**, 7971–7973 (1989).
